# Polychlorinated biphenyls, cytochrome P450 1A1 (*CYP1A1*) polymorphisms, and breast cancer risk among African American women and white women in North Carolina: a population-based case-control study

**DOI:** 10.1186/bcr941

**Published:** 2004-10-22

**Authors:** Yu Li, Robert C Millikan, Douglas A Bell, Lisa Cui, Chiu-Kit J Tse, Beth Newman, Kathleen Conway

**Affiliations:** 1 Department of Epidemiology, School of Public Heath, University of North Carolina, Chapel Hill, North Carolina, USA; 2 Lineberger Comprehensive Cancer Center, School of Medicine, University of North Carolina, Chapel Hill, North Carolina, USA; 3 National Institute of Environmental Health Sciences, Research Triangle Park, North Carolina, USA; 4 School of Public Health, Queensland University of Technology, Kelvin Grove, Queensland, Australia

**Keywords:** breast cancer, *CYP1A1*, polychlorinated biphenyls

## Abstract

**Introduction:**

Epidemiologic studies have not shown a strong relationship between blood levels of polychlorinated biphenyls (PCBs) and breast cancer risk. However, two recent studies showed a stronger association among postmenopausal white women with the inducible M2 polymorphism in the cytochrome P450 1A1 (*CYP1A1*) gene.

**Methods:**

In a population-based case-control study, we evaluated breast cancer risk in relation to PCBs and the *CYP1A1 *polymorphisms M1 (also known as CYP1A1*2A), M2 (CYP1A1*2C), M3 (CYP1A1*3), and M4 (CYP1A1*4). The study population consisted of 612 patients (242 African American, 370 white) and 599 controls (242 African American, 357 white).

**Results:**

There was no evidence of strong joint effects between *CYP1A1 *M1-containing genotypes and total PCBs in African American or white women. Statistically significant multiplicative interactions were observed between *CYP1A1 *M2-containing genotypes and elevated plasma total PCBs among white women (*P *value for likelihood ratio test = 0.02). Multiplicative interactions were also observed between *CYP1A1 *M3-containing genotypes and elevated total PCBs among African American women (*P *value for likelihood ratio test = 0.10).

**Conclusions:**

Our results confirm previous reports that *CYP1A1 *M2-containing genotypes modify the association between PCB exposure and risk of breast cancer. We present additional evidence suggesting that *CYP1A1 *M3-containing genotypes modify the effects of PCB exposure among African American women. Additional studies are warranted, and meta-analyses combining results across studies will be needed to generate more precise estimates of the joint effects of PCBs and *CYP1A1 *genotypes.

## Introduction

The role of organochlorine compounds such as polychlorinated biphenyls (PCBs) as etiologic agents for breast cancer has been investigated in several epidemiologic studies, but findings are inconsistent [1]. Initial studies examining breast tissue or serum showed significantly higher concentrations of PCBs in patients with breast cancer compared with controls [2-6], but more recent studies observed no association [7-10] or an inverse association [11] with breast cancer.

Several mechanisms have been proposed to explain how PCB exposure might increase the risk of breast cancer [12-15]. Owing to their lipophilic nature, organochlorine compounds undergo lifelong sequestration in animal and human adipose tissues. Adipose tissue within the human breast contains measurable levels of PCBs [16]. Metabolism of PCBs within breast tissue can generate reactive intermediates that cause oxidative damage and react with DNA. The proposed mechanism involves cytochrome P450 1A1 (*CYP1A1*), a gene that is inducible by PCBs [17,18]. *CYP1A1 *encodes aryl hydrocarbon hydrolase (AHH), an enzyme involved in the production of reactive epoxide intermediates from polycyclic aromatic hydrocarbons, steroid hormones, and other aromatic compounds [19]. On the basis of this model, individuals with higher *CYP1A1 *activity would be at increased risk of breast cancer when exposed to high levels of PCBs.

Several polymorphisms have been identified in *CYP1A1*, some of which lead to more highly inducible AHH activity [19-22]. *CYP1A1 *polymorphisms include M1 (T→C substitution at nucleotide 3801 in the 3'-noncoding region), M2 (A→G substitution at nucleotide 2455 leading to an amino acid change of isoleucine to valine at codon 462), M3 (T→C substitution at nucleotide 3205 in the 3'-noncoding region), and M4 (C→A substitution at nucleotide 2453 leading to an amino acid change of threonine to asparagine at codon 461). Studies of *CYP1A1 *in cultured human lymphocytes showed significantly elevated levels of inducible enzyme activity among persons with M2 genotypes [20,22]. Crofts and colleagues [21] reported that M2 alleles were associated with increased *CYP1A1 *inducibility at the mRNA level, and a threefold elevation in AHH enzyme activity. The M1 allele also encodes an inducible form of *CYP1A1 *[19,22,23].

Two recent epidemiologic studies showed an increased risk of breast cancer among postmenopausal white women with at least one M2 (valine) variant allele of *CYP1A1 *and a high serum level of PCBs [24,25]. These findings suggest that interactions between *CYP1A1 *polymorphisms and PCBs could be involved in the development of breast cancer, and genotyping for *CYP1A1 *could help to resolve inconsistencies in past epidemiologic studies. We used data from the Carolina Breast Cancer Study (CBCS), a population-based case-control study of African American and white women in North Carolina, to evaluate the effects of PCBs and *CYP1A1 *genotypes in relation to breast cancer risk.

## Materials and methods

### Study population

CBCS study participants were women aged 20–74 years residing in 24 contiguous counties in central and eastern North Carolina [26]. Patients were women with a first diagnosis of invasive breast cancer identified through a rapid ascertainment system implemented in cooperation with the North Carolina Central Cancer registry. Controls were selected from records of the North Carolina Division of Motor Vehicles and US Health Care Financing Administration, and were frequency-matched to patients on the basis of race and age. Information was collected through in-person interviews and included reproductive history, diet and lifestyle factors, a detailed family history of cancer, and occupational history. About 98% of participants who were interviewed agreed to give a 30 ml blood sample at the time of interview. Informed consent to obtain DNA and measurement of plasma concentration of PCBs was sought with the use of a form approved by the Institutional Review Board of the UNC School of Medicine in compliance with the Helsinki Declaration.

The CBCS was conducted in two phases. Phase I of the study was conducted between May 1993 and December 1996 [26]. Phase II of the study was conducted between 1996 and 2001. The total numbers of participants enrolled in Phase I of the CBCS were 861 patients (335 African American, 526 white) and 790 controls (332 African American and 526 white). The response rates were 76% for patients and 55% for controls. Genotyping for *CYP1A1 *was performed on the first 688 breast cancer patients and 702 controls enrolled in Phase I of the CBCS. Laboratory assays for organochlorines were conducted on blood samples from 748 patients and 659 controls in Phase I of the CBCS [26]. The present analyses were based on Phase I participants for whom both *CYP1A1 *genotyping and organochlorine measurements were available: 612 patients (242 African American, 370 white) and 599 controls (242 African American, 357 white).

### Laboratory methods

Methods for identification of plasma PCBs on CBCS plasma samples using gas chromatography/electron capture detection have been described previously [26]. Methods for genotyping of *CYP1A1 *on CBCS samples using restriction fragment length polymorphism–polymerase chain reaction have been described previously [27]. In a newly proposed nomenclature for *CYP1A1 *alleles, the M1 allele corresponds to CYP1A1*2A, M2 to CYP1A1*2C, M3 to CYP1A1*3, and M4 to CYP1A1*4 [28]. Data were missing on the following *CYP1A1 *genotypes due to unreadable gels or failure to amplify: M1 (10 patients and 7 controls), M2 (none missing), M3 (10 patients and 7 controls), and M4 (none missing). To correct for differences in plasma concentrations of chlorinated hydrocarbons attributable to blood lipids, the quantity of different lipid components in the plasma of each subject was measured with an automated enzymic assay, as described previously [26].

### Statistical methods

Imputed total PCB values were used in the data analyses. Imputation was performed by setting zero values and values below the detection limit (0.0125 ng/ml) to the detection limit divided by the square root of 2 (26). Hornung and Reed [29] recommended this method of imputation as an accurate approach for computing means and standard deviations for variables that include nondetectable values. In the present study, imputation did not affect the estimation of odds ratios (ORs) for breast cancer and total PCBs, because individuals in the lowest category (that is, below the median) remained in the lowest category after imputation. Lipid-adjusted total PCBs levels were calculated with Equation 2 of Phillips and colleagues [30]. Univariate analyses were performed for total PCBs, lipid-adjusted total PCBs, low to moderate chlorinated PCBs, highly chlorinated PCBs, and individual PCB congeners 99, 105, 118, and 153. Among controls in the CBCS, levels of individual PCB congeners and congener groups were highly intercorrelated [31]. ORs for the joint effects of *CYP1A1 *genotypes and PCB exposure did not differ substantially according to specific PCB congeners or congener groups, and ORs were similar to results obtained with total PCBs. We did not observe differences in ORs for breast cancer and PCB exposure, or the joint effects of *CYP1A1 *genotypes and PCBs, when we analyzed PCB congeners 99, 105, 118, or 153 alone, as suggested by Demers and colleagues [32]. Therefore, only results for lipid-adjusted total PCBs are presented.

Adjusted ORs for breast cancer and 95% confidence intervals (CIs) were calculated from unconditional logistic regression models. Total PCBs were categorized as greater than or equal to versus below the median, on the basis of the distribution in African American or white controls separately. PROC GENMOD of software package SAS (version 8.1; SAS Institute, Cary, NC) was used to incorporate offsets derived from the sampling probabilities used to identify eligible participants. Covariates included age, race (whites/African Americans), parity (nulliparous, 1 or ≥ 2), use of hormone replacement therapy (never or ever), oral contraceptive use (never or ever), breast feeding (never or ever), smoking (never, current, or former smoker), alcohol consumption (yes/no), family history of breast cancer (yes/no), benign breast biopsy (yes/no), income (less than $30,000/year or at least $30,000/year), and education (lower than high school, or high school and above). Continuous covariates, which included height, waist/hip ratio, and body mass index, were categorized on the basis of the median values of each variable in all controls.

ORs did not differ after adjusting for additional covariates, so results are presented for African American and white women adjusting for sampling fractions and age. Menopausal status was defined as described previously [27] and used to stratify analyses of *CYP1A1 *genotypes and PCB levels. Stratified analyses were also conducted on the basis of smoking history (ever or never). In addition, we estimated the increase in odds of breast cancer per 0.10 ng/ml increase in total PCB levels by coding lipid-adjusted total PCBs as a continuous variable, and stratifying on *CYP1A1 *genotypes. Linearity in the logit was tested using the Box–Tidwell transformation test as implemented in SAS (version 8.1). The assumption of linearity was not violated in premenopausal or postmenopausal African American or white women.

To assess the interaction on the additive scale between PCB levels and *CYP1A1 *genotypes, indicator variables were created for each category of joint exposure of PCBs and *CYP1A1 *genotype. Women with the homozygous common (non-M1, non-M2, non-M3, or non-M4) genotypes and the lowest level of exposure to PCBs were used as a common reference group. Interaction contrast ratios (ICRs) and 95% CIs were calculated for joint effects of the M1 genotype and PCBs [33]. ICR values greater than zero indicate greater than additive effects (synergy), and 95% CIs for the ICR that exclude zero can be used as a test for statistical significance at an alpha level of 0.05. To test for interaction on a multiplicative scale, likelihood ratio tests (LRTs) were conducted comparing logistic regression models with main effect terms for lipid-adjusted total PCBs and *CYP1A1 *genotypes and a product interaction term compared with models with main effects only. *P *values for LRTs less than 0.20 were considered statistically significant [34].

To test for independence of environmental exposure and genotype, means, medians, and distributions of total PCBs were determined in relation to *CYP1A1 *genotypes among African American and white controls. Medians for lipid-adjusted total PCBs were compared by using the Wilcoxon rank sum test. There were no statistically significant differences in medians for total PCBs comparing participants with *CYP1A1 *M1-containing versus non-M1-containing genotypes, M2 versus non-M2, M3 versus non-M3, or M4 versus non-M4 genotypes.

## Results

Genotype frequencies for *CYP1A1*, and ORs for breast cancer, have previously been reported for the CBCS [27]. In brief, genotype frequencies for *CYP1A1 *M1- and M3-containing genotypes were higher among African Americans than among whites, whereas M2- and M4-containing genotypes were more prevalent among whites. ORs for *CYP1A1 *genotypes and breast cancer were close to the null in African Americans and whites [27]. The association between plasma levels of total PCBs and breast cancer in the CBCS was reported previously [26]. A weak positive association for high levels of PCBs and breast cancer was found among African American women. ORs were close to the null for PCBs and breast cancer in white women.

ORs for breast cancer estimating the joint effects of total PCBs and *CYP1A1 *M1 genotypes on an additive scale are presented in Table 1. ORs were slightly elevated for PCB exposure greater than or equal to the median among premenopausal African American women regardless of *CYP1A1 *M1 genotype. There was no evidence for strong joint effects of PCBs and *CYP1A1 *M1-containing genotypes. ICRs were close to zero in African American and white women, and LRTs were not statistically significant. Joint effects for PCBs and *CYP1A1 *M2-containing genotypes among white women are presented in Table 2. Greater than additive joint effects were observed among all participants, with an ICR greater than zero and an associated *P *value of 0.03. Although imprecise, joint effects seemed to be stronger among premenopausal than among postmenopausal women. *P *values for LRTs were 0.02 among all participants, 0.007 among premenopausal women, and 0.66 among postmenopausal women.

ORs estimating the joint effects of PCBs and *CYP1A1 *M3 genotype among African American women are presented in Table 3. ORs were slightly elevated for women with elevated PCB levels and *CYP1A1 *M3-containing genotypes. An ICR greater than zero was observed in postmenopausal women. *P *values for LRTs were 0.10 in all participants, 0.71 among premenopausal women, and 0.11 in postmenopausal women. Joint effects of PCBs and *CYP1A1 *M4 genotypes among white women are presented in Table 4. An ICR greater than zero (*P *= 0.03) was observed among postmenopausal women. The *P *value for the LRT in postmenopausal women was 0.07. ICRs greater than zero for the joint effects of exposures with ORs less than 1.0 can be interpreted as antagonism [33]. Results were similar among smokers and non-smokers (data not shown).

ORs for breast cancer per 0.10 ng/ml increase in lipid-adjusted total PCBs showed only slight differences according to *CYP1A1 *M2 genotypes in white women. ORs for premenopausal women were 1.3 (95% CI 0.7–2.3) for those with *CYP1A1 *M2-containing genotypes and 1.0 (95% CI 0.9–1.1) for non-M2 genotypes. The corresponding ORs for postmenopausal women were 1.2 (95% CI 0.8–1.8) and 1.1 (1.0–1.2).

## Discussion

We estimated the joint effects of plasma levels of total PCBs and *CYP1A1 *genotypes in association with breast cancer, using previously collected data from a population-based case-control study of African American and white women in North Carolina. ORs were slightly elevated for premenopausal white women with *CYP1A1 *M2-containing genotypes and lipid-adjusted total PCBs greater than the median, and for postmenopausal African American women with M3-containing genotypes and total PCBs greater than the median. ORs and ICRs were imprecise because of small sample size, and many of our results could be due to chance. However, biologic evidence and previous epidemiologic studies support the possibility of causal interaction between *CYP1A1 *genotypes (in particular, M2-containing genotypes) and PCB exposure in the etiology of breast cancer.

PCBs are metabolized by cytochrome P450 enzymes, activate *CYP1A1*, and produce free-radical-induced oxidative DNA damage in breast tissue [35]. With regard to *CYP1A1 *M1-containing genotypes, a previous study by Laden and colleagues [25] did not report a positive association for M1-containing genotypes and high levels of PCBs in postmenopausal women: the OR for women in the highest one-third of lipid-adjusted total PCBs (at least 0.67 μg per g lipid) and M1-containing genotypes was 1.1 (95% CI 0.5–2.5) compared with women in the lowest one-third of PCBs with non-M1-containing genotypes. In our data set, the corresponding age-adjusted OR for lipid-adjusted total PCBs of 0.67 ng/ml or more and *CYP1A1 *M1-containing genotypes among postmenopausal white women was 1.8 (95% CI 0.5–6.9).

With regard to *CYP1A1 *M2-containing genotypes, Moysich and colleagues [24] reported an OR for breast cancer of 2.9 (95% CI 1.2–7.5) in postmenopausal women with total PCBs between 3.72 and 19.04 ng/g and *CYP1A1 *M2-containing genotypes compared with women with low PCBs and non-M2-containing genotypes. In our data set, the corresponding age-adjusted OR for total PCBs of 3.73 ng/ml or more (not lipid-adjusted) and *CYP1A1 *M2-containing genotypes was 6.3 (95% CI 0.7–55.0). Laden and colleagues [25] reported an OR of 2.8 (95% CI 1.0–7.8) for women with the highest one-third of lipid-adjusted total PCBs (0.67 μg per g lipid) and *CYP1A1 *M2-containing genotypes, compared with women in the lowest one-third of PCBs and non-M2-containing genotypes. The corresponding OR in our data set for lipid-adjusted total PCBs ≥ 0.67 ng/ml and *CYP1A1 *M2-containing genotypes was 4.7 (95% CI 0.5–43.1). It would be helpful to combine individual-level results across these three studies to obtain more precise estimates of the joint effects of *CYP1A1 *genotypes and high levels of PCB exposure.

A strength of our study is the fact that we included both African American and white women, and we examined the effects of the four known *CYP1A1 *alleles: M1, M2, M3, and M4. Previous studies [24,25] included only white women and did not estimate ORs for *CYP1A1 *M3- or M4-containing genotypes. In previous analyses of this data set [26], African American women showed higher plasma levels of PCBs and a stronger relationship between total PCBs and breast cancer than that in white women. Our results suggest that further study of *CYP1A1 *M3-containing genotypes in African American women is warranted, particularly in combination with PCB exposure. Another strength of our study is that fact that we estimated joint effects for specific PCB congener groups and *CYP1A1 *genotypes. PCB congener groups may differ in biologic activity in ways that are relevant to breast cancer etiology [35-39]. However, because of the strong intercorrelation of specific congeners with total PCBs, we were unable to distinguish strong congener-specific effects.

A weakness of our study was the fact that, because of the small sample size, we were unable to estimate ORs with precision in all of the subgroups of interest. Moysich and colleagues [24] reported that breast cancer risk was significantly increased among women with elevated PCB body burden and *CYP1A1 *M2 genotypes who had ever smoked cigarettes. Laden and colleagues [25] observed similar results. We did not observe differences in ORs for the joint effects of total PCBs and *CYP1A1 *genotypes according to smoking status. However, we lacked power to address the effects of smoking dose or duration. ORs were imprecise when we subdivided study participants on the basis of menopausal status, and any differences could be due to chance. Owing to the case-control study design, patients were enrolled after diagnosis of breast cancer. As described previously [26], we did not observe evidence for disease-related changes in plasma organochlorine levels when we adjusted for weight loss or gain and stage at diagnosis. Limited information on diet was collected in this study. We did not observe correlations between total PCB levels and the consumption of fruits, vegetables, or fish (data not shown), but the confounding effects of other dietary exposures cannot be ruled out.

## Conclusions

Data from the CBCS provides evidence that subgroups of women with *CYP1A1 *M2 and M3 polymorphisms and high levels of PCB exposure might have a modestly elevated risk of breast cancer. Our results confirm findings from previous studies with respect to the highly inducible *CYP1A1 *M2 genotype, and suggest that M3-containing genotypes might also modify risk of breast cancer associated with PCB exposure in African American women. Additional studies with a large sample size are warranted to confirm or refute these findings. In addition, meta-analyses combining individual-level data from epidemiologic studies of *CYP1A1 *and breast cancer will be needed to generate more precise estimates of the joint effects of PCBs and *CYP1A1 *genotypes.

## Abbreviations

AHH = aryl hydrocarbon hydrolase; CBCS = Carolina Breast Cancer Study; CI = confidence interval; *CYP1A1 *= cytochrome P450 1A1; ICR = interaction contrast ratio; LRT = likelihood ratio test; OR = odds ratio; PCBs = polychlorinated biphenyls.

## Competing interests

The author(s) declare that they have no competing interests.

## Authors' contributions

YL and LC conducted the laboratory analyses; YL, RM, and C-KT conducted the statistical analyses; YL, RM, DB, LC, C-KT, BN and KC participated in writing the manuscript.

## Figures and Tables

**Table 1:** Odds ratios for breast cancer and total PCBs in relation to *CYP1A1 *M1 genotypes in African American and white women

Total PCBs^a^	*CYP1A1 *M1 genotype	All participants		Premenopausal		Postmenopausal	
		Patients/controls	OR (95% CI)^b^	Patients/controls	OR (95% CI)^b^	Patients/controls	OR (95% CI)^b^

African Americans							
<0.430	Non-M1	66/75	Referent	46/51	Referent	20/24	Referent
≥0.430	Non-M1	75/67	1.5 (0.9–2.5)	21/16	1.9 (0.9–4.2)	54/51	1.0 (0.5–2.3)
<0.430	Any M1	42/46	1.0 (0.6–1.7)	35/33	1.2 (0.6–2.2)	7/13	0.6 (0.2–1.8)
≥0.430	Any M1	59/54	1.4 (0.8–2.5)	17/14	1.7 (0.8–4.1)	42/40	1.0 (0.5–2.3)
ICR (95% CI)			0.0 (-0.9,0.9)		-0.3 (-2.2,1.6)		0.4 (-0.5,1.3)
Whites							
<0.349	Non-M1	174/133	Referent	118/83	Referent	56/50	Referent
≥0.349	Non-M1	122 148	0.7 (0.5–1.0)	38/43	0.6 (0.4–1.1)	84/105	0.8 (0.5–1.3)
<0.349	Any M1	45/44	0.8 (0.5–1.2)	29/31	0.7 (0.4–1.2)	16/13	1.1 (0.5–2.5)
≥0.349	Any M1	29/32	0.8 (0.4–1.4)	11/10	0.7 (0.3–1.9)	18/22	0.8 (0.4–1.7)
ICR (95% CI)			0.4 (-0.2,0.9)		0.5 (-0.3,1.2)		0.0 (-1.1,1.0)

^a^Lipid-adjusted total imputed PCBs (ng/ml).

^b^Odds ratios adjusted for age and sampling fractions.

Any M1, M1/M1 or M1/non-M1; CI, confidence interval; ICR, interaction contrast ratio; non-M1, non-M1/non-M1.

**Table 2:** Odds ratios for breast cancer and total PCBs in relation to *CYP1A1 *M2 genotypes in white women

Total PCBs^a^	*CYP1A1 *M2 genotype	All participants		Premenopausal		Postmenopausal	
		Patients/controls	OR (95% CI)^b^	Patients/controls	OR (95% CI)^b^	Patients/controls	OR (95% CI)^b^

<0.349	Non-M2	210/158	Referent	141/98	Referent	69/60	Referent
≥0.349	Non-M2	138/166	0.7 (0.5–1.0)	45/51	0.6 (0.4–1.0)	93/115	0.8 (0.5–1.2)
<0.349	Any M2	11/20	0.4 (0.2–0.8)	8/16	0.3 (0.1–0.8)	3/4	0.7 (0.1–3.1)
≥0.349	Any M2	15/14	0.9 (0.4–1.9)	6/2	2.1 (0.4–10.6)	9/12	0.7 (0.3–1.9)
ICR (95% CI)			0.8 (0.1, 1.6)		2.1 (-1.3,5.5)		0.3 (-0.9,1.5)

^a^Lipid-adjusted total imputed PCBs (ng/ml).

^b^Odds ratios adjusted for age and sampling fractions.

Any M1, M1/M1 or M1/non-M1; CI, confidence interval; ICR, interaction contrast ratio; non-M1, non-M1/non-M1.

**Table 3:** Odds ratios for breast cancer and total PCBs in relation to *CYP1A1 *M3 genotypes in African American women

Total PCBs^a^	*CYP1A1 *M3 genotype	All participants		Premenopausal		Postmenopausal	
		Patients/controls	OR (95% CI)^b^	Patients/controls	OR (95% CI)^b^	Patients/controls	OR (95% CI)^b^

<0.430	Non-M3	95/95	Referent	73/70	Referent	22/25	Referent
≥0.430	Non-M3	105/100	1.3 (0.8–2.0)	31/24	1.6 (0.8–3.2)	74/76	1.0 (0.5–2.0)
<0.430	Any M3	13/26	0.6 (0.3–1.2)	8/14	0.7 (0.3–1.7)	5/12	0.5 (0.2–1.6)
≥0.430	Any M3	29/21	1.6 (0.8–3.2)	7/6	1.5 (0.5–4.7)	22/15	1.5 (0.6–3.6)
ICR (95% CI)			0.8 (-0.3,1.9)		0.2 (-1.7,2.1)		1.0 (-0.2,2.1)

^a^Lipid-adjusted total imputed PCBs (ng/ml).

^b^Odds ratios adjusted for age and sampling fractions.

Any M1, M1/M1 or M1/non-M1; CI, confidence interval; ICR, interaction contrast ratio; non-M1, non-M1/non-M1.

**Table 4:** Odds ratios for breast cancer and total PCBs in relation to *CYP1A1 *M4 genotypes in white women

Total PCBs^a^	*CYP1A1 *M4 genotype	All participants		Premenopausal		Postmenopausal	
		Patients/controls	OR (95% CI)^b^	Patients/controls	OR (95% CI)^b^	Patients/controls	OR (95% CI)^b^

<0.349	Non-M4	202/159	Referent	132/103	Referent	70/56	Referent
≥0.349	Non-M4	139/163	0.7 (0.5–1.0)	46/48	0.8 (0.5–1.2)	93/115	0.7 (0.4–1.1)
<0.349	Any M4	19/19	0.8 (0.4–1.6)	17/11	1.2 (0.5–2.7)	2/8	0.2 (0.0–0.9)
≥0.349	Any M4	14/17	0.7 (0.3–1.6)	5/5	0.8 (0.2–2.9)	9/12	0.7 (0.3–1.7)
ICR (95% CI)			0.2 (-0.6,1.0)		-0.1 (-1.6,1.3)		0.8 (0.1,1.5)

^a^Lipid-adjusted total imputed PCBs (ng/ml).

^b^Odds ratios adjusted for age and sampling fractions.

Any M1, M1/M1 or M1/non-M1; CI, confidence interval; ICR, interaction contrast ratio; non-M1, non-M1/non-M1.
